# Hyperoxia toxicity after cardiac arrest: What is the evidence?

**DOI:** 10.1186/s13613-016-0126-8

**Published:** 2016-03-22

**Authors:** Jean-François Llitjos, Jean-Paul Mira, Jacques Duranteau, Alain Cariou

**Affiliations:** Medical Intensive Care Unit, Cochin Hospital, Hôpitaux Universitaires Paris Centre, Assistance Publique des Hôpitaux de Paris, 27 rue du Faubourg Saint-Jacques, 75014 Paris, France; Faculté de Médecine, Université Paris Descartes, Sorbonne Paris Cité, 15 rue de l’école de Médecine, 75006 Paris, France; Anesthesia and Intensive Care Department, Bicêtre Hospital, Assistance Publique des Hôpitaux de Paris, 94275 Le Kremlin-Bicêtre, France; Université Paris Sud XI, Orsay, France

**Keywords:** Hyperoxia, Cardiac arrest, Cardiopulmonary resuscitation, Ischemia reperfusion, Oxidative stress, Reactive oxygen species

## Abstract

This review gives an overview of current knowledge on hyperoxia pathophysiology and examines experimental and human evidence of hyperoxia effects after cardiac arrest. Oxygen plays a pivotal role in critical care management as a lifesaving therapy through the compensation of the imbalance between oxygen requirements and supply. However, growing evidence sustains the hypothesis of reactive oxygen species overproduction-mediated toxicity during hyperoxia, thus exacerbating organ failure by various oxidative cellular injuries. In the cardiac arrest context, evidence of hyperoxia effects on outcome is fairly conflicting. Although prospective data are lacking, retrospective studies and meta-analysis suggest that hyperoxia could be associated with an increased mortality. However, data originate from retrospective, heterogeneous and inconsistent studies presenting various biases that are detailed in this review. Therefore, after an original and detailed analysis of all experimental and clinical studies, we herein provide new ideas and concepts that could participate to improve knowledge on oxygen toxicity and help in developing further prospective controlled randomized trials on this topic. Up to now, the strategy recommended by international guidelines on cardiac arrest (i.e., targeting an oxyhemoglobin saturation of 94–98 %) should be applied in order to avoid deleterious hypoxia and potent hyperoxia.

## Background

Oxygen has a pivotal role in medicine as a lifesaving therapy in many emergency situations. In order to avoid hypoxia-related mortality and morbidity, oxygen is delivered in acute care situations in a liberal way, even if hypoxia is not confirmed. However, as every medication, experimental and clinical studies have highlighted some physiological potent side effects of high oxygen tension that could worsen outcome [1]. Cardiac arrest is the archetypal situation given the imperious need of rapid oxygen delivery in organs. Nevertheless, this global ischemia–reperfusion syndrome produces high amounts of reactive oxygen species that could be significantly increased by high oxygen tension. Thus, hyperoxia in the post-resuscitation context of cardiac arrest is an important topic. Despite several experimental and clinical studies, this subject remains at the center of conflicting results with insufficient body of evidences. We screened PubMed, Embase and Cochrane databases using the following keywords with various combinations: “cardiac arrest,” “oxygen,” “oxidative stress” and “hyperoxia.” Pediatric data concerning oxygen management during and after cardiac arrest were neither listed nor analyzed given differences in etiologies, management and outcome between adult and pediatric patients resuscitated from cardiac arrest. We herein provide an overview of the present knowledge of the pathophysiological effects of oxygen, review experimental and clinical studies and develop some concepts that could be beneficial for further studies.

## Pathophysiology of oxygen and hyperoxia

Hyperoxia occurs when the partial pressure of intraalveolar oxygen exceeds normal breathing conditions, thus leading to hyperoxemia, which is defined by an increased arterial O_2_ partial pressure. Oxygen concentration in the blood is a combination of 3 main parameters and the sum of the dissolved oxygen and the hemoglobin-bound oxygen, defined by the following equation:

$$\begin{aligned} {\text{OBC}} & = \left[ {{\text{Hemoglobin}} - {\text{bound}}\;{\text{oxygen}}} \right] \\&\quad + \left[ {{\text{Dissolved}}\;{\text{oxygen}}} \right] \\ {\text{OBC}} & = \left( {1.34 \times \left[ {\text{Hb}} \right] \times \left[ {{\text{SaO}}_{2} } \right]} \right) + \left( {{\text{Kh}} \times \left[ {{\text{PaO}}_{2} } \right]} \right) \\ \end{aligned}$$

where OBC: oxygen blood concentration (mL of oxygen per liter of blood); 1.34: oxygen-carrying capacity of hemoglobin (mL of oxygen per gram of hemoglobin), [Hb]: hemoglobin, [SaO_2_]: hemoglobin oxygen saturation, [Kh]: solubility coefficient of oxygen, [PaO_2_]: arterial oxygen partial pressure.

**Fig. 1:**
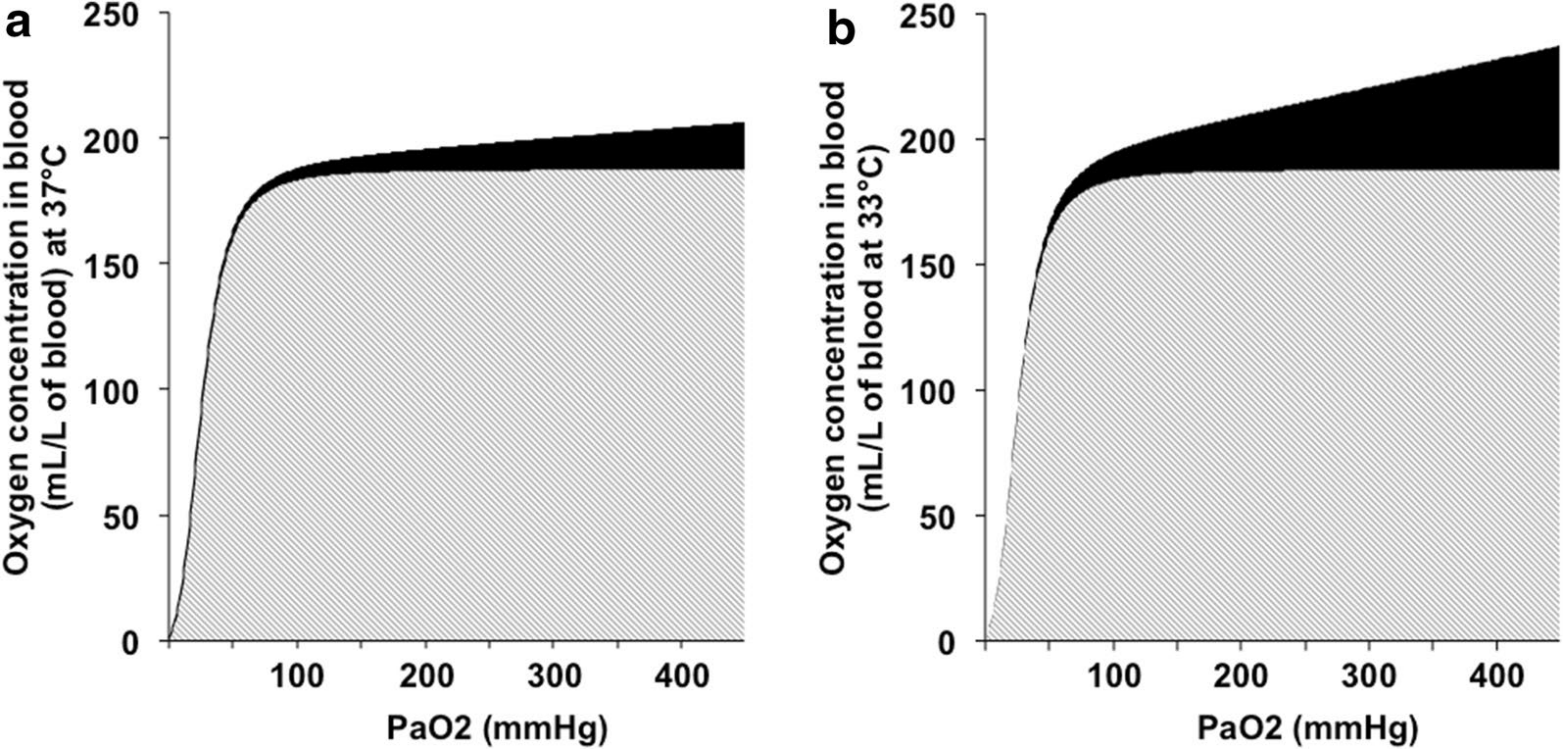
Hypothermia increases quantity of dissolved oxygen in blood. (**a**) + (**b**) The *gray area* under the *curve* represents amounts of hemoglobin-bound oxygen, and the *black area* under the *curve* represents quantity of dissolved oxygen. If a 33 °C temperature is associated with a leftward shift of the oxyhemoglobin *curve* when compared to a 37 °C central temperature, hypothermia (**b**) increases dissolved oxygen quantity in blood. For instance, there is a 2.7-fold increase in dissolved quantity of oxygen between 33 and 37 °C

At normal pH and normal temperature, increased O_2_ breathing raises the amount of dissolved O_2_ without modifying the close to 100 % hemoglobin saturation given the sigmoid-shaped hemoglobin to oxygen dissociation curve (Fig. 1). If the Henry’s law states a linear relation between arterial oxygen partial pressure and oxygen solubility, temperature is a main parameter influencing the solubility coefficient parameter *Kh*. For instance, using the work by Battino et al. [2] and the Van’t Hoff equation, *Kh* (37 °C) = 0.0031 whereas *Kh* (33 °C) = 0.0084. Therefore, hypothermia increases amounts of oxygen dissolved in the blood (Fig. 1). Moreover, several factors affect the dissociation curve of oxyhemoglobin such as temperature, pH (Borr effect), PaCO_2_ (Haldane effect) and 2,3-biphosphoglyceric acid [3]. A left shift of the curve that means a higher oxygen affinity of hemoglobin is induced by hypothermia, hypocarbia and alkalosis. This arterial accumulation of dissolved oxygen is supposed to exert deleterious effects through various mechanisms that are intricately linked: reactive oxygen species (ROS) overproduction, pulmonary toxicity, cardiac and neurological affects.

ROS are unstable and highly reactive molecules, participating in a broad spectrum of cellular events such as production of inflammation mediators, intracellular messengers or anti-infectious effectors. In mammalian, ROS can be of endogenous or exogenous origin (radiation, pollution, drugs, medication, smoking). Under physiological condition, ROS are produced by the respiratory chain in mitochondria or by enzymatic reactions.

Toxicity of ROS consists essentially in lipid peroxidation, protein oxidation and DNA damages. Lipid peroxidation, when affecting intra- or extracellular membranes, leads to enzyme inactivation, thiols oxidation and mitochondrial respiratory chain inhibition [4]. Protein oxidation confers resistance to proteolysis, mostly by aggregation [5]. Toxicity of ROS with regard to DNA is dominated by cellular cycle modification, apoptosis and carcinogenesis [6].

The defenses developed to minimize, prevent and repair injuries caused by oxidative stress include enzymatic ROS removal (superoxide dismutase, catalase and glutathione peroxidase) and the non-enzymatic quenching of ROS by antioxidant (glutathione, albumin, A and E vitamin and thiols) [7]. Mechanisms involved in cell death induced by high oxygen tension include apoptosis, necrosis or mixed-mechanisms phenotype, depending on the cell type investigated. As oxygen is one of the main modulating factors of ROS production, hyperoxia appears to be a major provider of ROS overproduction, particularly in the inflammatory context of cardiac arrest, thus leading to an imbalance in the oxidative status.

Hyperoxia is known to exert toxic pulmonary effects through pulmonary gas exchange impairment or direct pulmonary toxicity. The alteration in gas exchanges is driven by the inhibition of hypoxic pulmonary vasoconstriction and by the “adsorption atelectasis” that increases intrapulmonary right-to-left shunt. Direct pulmonary toxicity, so-called *Lorrain*–*Smith* effect, consists in ROS-related direct toxicity on alveolar capillary barrier and leads to lung passageways congestion and hemorrhagic pulmonary edema [8].

Hyperoxia decreases cardiac output due to both a drop of heart rate [9] and a rise in vascular resistance [10]. Supra-physiological oxygen tensions also alter the microperfusion through a decreased capillary perfusion [11] and the systemic perfusion. This hyperoxia-induced vasoconstriction may result from a fall in NO bioavailability [12], with a potential contribution of ROS [13].

Hyperoxia possibly has toxic effects on the central nervous system, the so-called *Paul Bert* effect, which could reach its climax with tonic–clonic seizures [14]. This deleterious effect is particularly reported to occur in supra-atmospheric pressure such as hyperbaric chambers or diving and possibly related to ROS formation [15].

## Experimental evidences in the cardiac arrest context

### Various models with significant disparities

Various evidences from animal studies sustain the rationale for brain lesions after hyperoxic resuscitation. These studies compare the administration of hyperoxic to lower concentration of oxygen following resuscitation on neurological, histological and neurochemical outcome (Table 1). However, these studies show significant disparities. First, experimental models use three different animals (i.e., dogs, rats and pigs) with various resuscitation protocols. Second, even if most of the cardiac arrests are induced by electrically induced ventricular fibrillation, diverse cardiac arrest models are used. For instance, the use of asphyxia by Lipinski et al. may strongly influence response to oxygen during and after cardiopulmonary resuscitation. Thus, given the differences in animal species and cardiac arrest models, Pilcher et al. recently performed a meta-analysis of several studies in order to evaluate effects of hyperoxia on neurological outcome after cardiac arrest [16]. Meta-analysis of six studies with 95 animals revealed that 100 % FiO_2_ is associated with worse neurological outcome with a standardized mean difference of −0.64 (95 % CI −1.06 to −0.22). However, this result should be considered with caution given the heterogeneity of models and the small size of the overall population.

**Table 1:** Experimental studies evaluating effects of high oxygen tensions in the cardiac arrest context

Study (year)	Animal (*n*=)	Mechanism of arrest	Arrest duration (min)	Inspired oxygen during CPR	Main outcome measure	Follow-up period after ROSC	Inspired oxygen after ROSC	Main result
Zwemer (1994)	Dogs (27)	Electrical	9	21 versus 100 %	Neurological (score)	24 h	21 versus 100 % during 1 h then room air during 24 h	Worse neurological outcome after hyperoxia
Marsala (1992)	Dogs (13)	Intracardial KCl bolus	15	21 versus 100 %	Neurological (histological)	1 h	21 versus 100 %	Increased neuron vulnerability after hyperoxia
Zwemer (1995)	Dogs (17)	Electrical	9	8.5 versus 12 versus 21 % during 15 min	Mortality and neurological (score)	24 h	Room air 15 min after CPR	Worse neurological outcome and increased mortality after hypoxia
Liu (1998)	Dogs (20)	Electrical	10	21 versus 100 %	Neurological (score) and oxidative stress (lipid peroxidation)	24 h	21–30 versus 100 %	Worse neurological outcome and increased oxidative stress after hyperoxia
Lipinski (1998)	Rats (22)	Asphyxia	5–8	21 versus 100 %	Neurological (score)	24, 48, 72 h	21 versus 100 % during 1 h then room air	No differences
Rosenthal (2003)	Dogs (9)	Electrical	10	Hyperbaric oxygenation versus 21 %	Neurological (score and histological)	24 h	Hyperbaric oxygenation versus 21 %	Hyperbaric oxygenation improved neurological outcome
Vereczki (2006)	Dogs (12)	Electrical	10	21 versus 100 %	Neurological (score) and oxidative stress (pyruvate dehydrogenase)	24 h	100 % for 1 h versus 21 % then PaO_2_ [80–100] in both groups	Increased oxidative stress and delayed neuronal death
Balan (2006)	Dogs (17)	Electrical	10	100 %	Neurological (score and histological)	24 h	100 % for 1 h versus 21–30 % with titration	Oxymetry-guided reoxygenation improved neurological score and decreased neuronal death
Richards (2006)	Dogs (16)	Electrical	10	21 versus 100 %	Antioxidative enzyme (pyruvate dehydrogenase)	2 h	100 % for 1 h versus 21–30 % with titration	Hyperoxia impairs oxidative stress metabolism
Richards (2007)	Dogs (13)	Electrical	10	21 versus 100 %	Oxidative stress (glutamate)	2 h	100 % for 1 h versus 21–30 % with titration	Hyperoxia impairs oxidative metabolism
Yeh (2009)	Rats (23)	Intravenous KCl bolus	6	0 versus 21 versus 100 %	Neurological (score)	1 h	0 versus 21 versus 100 % during 2 min then all animals with 100 %	CPR ventilation without oxygen worsen neurological score
Bruchen (2010)	Pig (15)	Electrical	8	100 %	Neurological (score and histological)	5 days	100 % during 10 min versus 60 min	Prolonged hyperoxia aggravated neurological outcome
Angelos (2011)	Rats (?)	Intravenous KCl bolus	6.5	21 %	Heart mitochondrial respiratory function	1 h	40 versus 100 % for 60 min	Hyperoxia impaired heart mitochondrial function

### A body of evidence highlighting the role of oxidative stress

Within a broad spectrum of cellular events, impaired cerebral enzymes appear to play a pivotal role in ischemia–reperfusion brain injury by oxidative molecular mechanisms. Instead of producing amounts of useful aerobic energy metabolites, anaerobic glycolysis observed during ischemia reperfusion condition produces excessive lactate, thus decreasing ATP production. Vereczki et al. investigated the effects of normoxic resuscitation on loss of pyruvate dehydrogenase enzyme (which induces the decarboxylation of pyruvate into NADH and acetyl coenzyme A) and neuronal death using a dog model of electrical cardiac arrest. The post-cardiac arrest hyperoxic ventilation led to higher loss of pyruvate dehydrogenase enzyme and to an increase in hippocampal neuronal death [17]. To go further, Richards et al. examined the hypothesis that the initial hippocampal neuronal loss could be related to a preferential decrease in aerobic metabolism when compared to the cortex. They investigated the pyruvate dehydrogenase enzyme activity decrease under hyperoxic condition into an electrical ventricular fibrillation dog model of cardiac arrest. Using carbon isotypes inclusion and spectroscopy, they found that dogs resuscitated with high levels of oxygen (100 vs. 21–30 % of inspired oxygen) had a decrease in PDH activity [18, 19]. This study seems to suggest a relation between increased oxidative stress and hyperoxia resuscitation in cardiac arrest.

### Relevance of experimental models

Most of all, experimental studies are not a good reflection of current standard of care in cardiopulmonary resuscitation. First, some animals are anesthetized and mechanically ventilated before cardiac arrest. Therefore, hyperoxia is sometimes induced prior to cardiac arrest and administration of high-inspired oxygen fraction has an impact on further analysis. Second, whereas temperature is nowadays widely admitted to influence oxygenation parameters, no study is performed under therapeutic hypothermia and central body temperature management details are scarce. Third, neurological examination is performed in a time frame ranging from 2 h to 5 days. Indeed, data are lacking on neurological long-term outcome.

Preclinical data available in animals are characterized by a large disparity in species, in arrest mechanisms and in resuscitation protocols. Furthermore, the discrepancy between recorded effects of hyperoxia on neurological outcome does not provide a clear answer. Mechanisms need to be elucidated, particularly the relationship between neurological damages following resuscitation and ROS overproduction within cerebral tissue under hyperoxic conditions. Finally, the clinical significance of these different experimental models studies is unclear.

## Hyperoxia and cardiac arrest in humans

Based on the observational data from Norway that report a better outcome in centers that did not use hyperoxygenation during CPR [20], the hypothesis of the neurological detrimental effects of hyperoxia after cardiopulmonary resuscitation was first investigated prospectively in a small study including 28 patients, randomized to be ventilated with either 30 or 100 % inspired oxygen after out-of-hospital cardiac arrest. Using neuron-specific enolase (NSE) 24 h after resuscitation as a specific marker of neuronal injury, the authors found a statistically significant increase in this enzyme within a subgroup of patients not treated with therapeutic hypothermia, thus suggesting both neurological deleterious effects of hyperoxia and putative beneficial effects of hypothermia on hyperoxia damages [21]. Regrettably, this trial does not incorporate enough patients to appraise neurological outcome or survival. Moreover, a recent randomized controlled feasibility trial failed to safely titrate oxygen in the pre-hospital period [22]. Therefore, most of the data are provided by retrospective studies (Table 2).

**Table 2:** Human studies evaluating effects of hyperoxia in the cardiac arrest context

Study (year)	Study period	Patients (*n*=)/hyperoxia prevalence (%)	Shockable rhythm (%)	Blood gas analysis timing	Hyperoxia definition	In-hospital/hyperoxia mortality (%)	Comparison group	Therapeutic hypothermia	Main outcome measure	In-hospital mortality (OR, 95 % CI)	Poor neurological status (OR, 95 % CI)
Kilgannon (2010)	2001/2005	6326/18	NA	First PaO_2_	>300 mmHg	56/63	Normoxia	6 % <34 °C during first 24 h	In-hospital mortality	1.8 (1.5–2.2)	?
Bellomo (2011)	2000/2009	12,108/10	NA	Lower PaO_2_	>300 mmHg	58/59	Normoxia	33 % <34 °C during first 24 h	In-hospital mortality	1.2 (1.0–1.5)	?
Kilgannon (2011)	2001/2005	4459/NA	NA	Highest PaO_2_	Continuous variable	54/?	Normoxia	6 % <34 °C during first 24 h	In-hospital mortality	1.69 (1.56–2.07)	?
Janz (2012)	2007/2012	170/about 30	61	Highest PaO_2_	Continuous variable	55/?	Normoxia	100 % <33 °C	In-hospital mortality	1.4 (1–2)	1.48 (1.03–2.13)
Ihle (2013)	2007/2011	584/6	100	Worst PaO_2_	>300 mmHg	42/47 %	Normoxia	?	In-hospital mortality	1.2 (0.52–2.82)	?
Nelskyla (2013)	2008/2010	119/41	40	Highest PaO_2_	>300 mmHg	63/59	Non-hyperoxia	30 %	Factors associated with hyperoxia exposure	0.76 (0.36–1.61)	?
Spindelboeck (2013)	2003/2010	145/20	?	<60 min after CPR	>300 mmHg	?/?	Non-hyperoxia	?	Rates of hospital admission	?	?
Vaahersalo (2014)	2010/2011	409/NA	60	Mean 24 h PaO_2_ level	Continuous variable	45/?	Non-hyperoxia	71 %	CPC at 12 months	?	1 (0.99–1.01)
Lee (2014)	2008/2012	213/1.1	25	Mean PaO_2_ in 8 ABG	Quartiles of PaO_2_	29.6/?	PaO_2_ [116–134]	100 % <33 °C	In-hospital mortality	0.65 (0.22–1.85)	4.22 (1.22–14.58)
Elmer (2015)	2008/2010	184/36	38	Time spent within each PaO_2_ level	Continuous variable	54?/?	Non-hyperoxia	66 % <33 °C	Survival to hospital discharge	0.83 (0.69–0.99) per hour	0.8 (0.3–2.13)
Helmerhorst (2015)	2007/2012	5258/2.7	?	Lowest PaO_2_/FiO_2_ in first 24 h	>300 mmHg	53.9/?	Normoxia	80 % <34 °C	In-hospital mortality	1.13 (0.81–1.57)	?

### Two main retrospective studies with conflicting results

Two recently published articles mainly dominate retrospective studies in humans. First, Kilgannon et al. reported a retrospective cohort of patients extracted from an American database consolidating 120 centers (Project IMPACT) [23]. In multivariate analysis, exposition to hyperoxia on mortality is associated with an odds ratio of 1.8 (95 % CI 1.5–2.2). On the other hand, Bellomo et al. reported a retrospective cohort of patients extracted from an Australian and New Zealand database clustering 125 centers [24]. In multivariate analysis, exposition to hyperoxia was associated with an odds ratio of 1.2 (95 % CI 1.1–1.6), but no longer using a Cox proportional hazard model and when adjusted on FiO_2_. Major differences in the definition and in the analysis of hyperoxia may explain these conflicting results.

### An important variability in hyperoxia definition

In order to ensure comparison after the work by Kilgannon et al., most of the studies define hyperoxia as a PaO_2_ higher or equal to 300 mmHg. This definition is based a priori and arbitrarily on an experimental study evaluating the effect of hypoxemic reperfusion on brain histopathological changes in the pig [25]. There is no physiological evidence in humans to support the use of this cutoff value, and the use of a single value may under or overestimate hyperoxia incidence. Moreover, a definition of groups using PaO_2_ presupposes a threshold effect of oxygen and eliminates a dose-dependant effect. This point has been investigated in three studies. Analyzing PaO_2_ as a continuous variable, Kilgannon et al. and Janz et al. found in multivariate analysis that high levels of PaO_2_ were associated with an increased mortality (OR 1.69; 95 % CI 1.56–2.07 and OR 1.4; 95 % CI 1.02–2.01, respectively) [26, 27]. Interestingly, these two studies had similar overall mortality (54 and 55 %, respectively). The third study found no association between PaO_2_ levels of exposure and the neurological status at 12 months as the main outcome measure [28]. These results support the hypothesis of a dose-dependant association between supra-normal oxygen tension and outcome. However, various precisions are lacking in the work done by Kilgannon et al., for instance cardiac arrest precisions (according to Utstein style) like the cause of death, neurological outcome or ventilation parameters, and thus may participate to overestimation of oxygen role in mortality.

In addition, the value of PaO_2_ of interest is not clearly defined in the literature. In the first study published by Kilgannon et al., hyperoxia was defined according to the first blood gas analysis available within the first 24 h after intensive care unit admission; some studies analyze the highest PaO_2_ in the first 24 h [26, 27, 29], whereas in the studies reported by Bellomo et al. and Ihle et al. hyperoxia was defined using the “worst” blood gas analysis within the first 24 h [24, 30]. These variations seem to affect hyperoxia prevalence, which is, for instance, ranging in all studies from 2.7 to 41 % and may thus introduce bias for further analysis on mortality.

### Influence of hypothermia on oxygen status during post-cardiac arrest resuscitation

It is now well established that temperature mainly influences ventilation and oxygenation parameters in patients. Hypothermia induces a right shift of the oxygen dissociation curve, decreases oxygen amounts released and increases carbon dioxide solubility [31]. Precisions on temperature are lacking in some studies [30, 32], whereas various proportions of patients are treated with therapeutic hypothermia in others (even if the induced or spontaneous nature of this hypothermia is not clearly indicated) ranging from 6 % [23] to 80 % [33]. Moreover, methodological details regarding temperature corrections of arterial blood gas are missing in most of the studies. To address this concern, two studies investigated effects of oxygenation status on mortality under hypothermia conditions. Once again, analysis of PaO_2_ differs between these two studies and thus limits comparison: Janz et al. use the highest PaO_2_ [27], whereas Lee et al. use the mean PaO_2_ using 8 arterial blood gas from ROSC to rewarming [34]. Therefore, using the previously reported cutoff value of 300 mmHg, 1.1 % of patients presented hyperthermia in the study by Lee et al. while about 30 % of patients were in the hyperoxia group in the study by Janz et al. This issue may have a major impact on overall mortality in most of the studies given the various proportions of patients under hypothermia. For instance, difference in mortality reported by Kilgannon et al. may be overestimated given the 6 % of patients with a central temperature under 34 °C within the first 24 h when compared to the 66 % of patients under the same condition in the work by Elmer et al. [35].

### Oxygen exposition after cardiac arrest: the crucial period

Another problem of interest is the time point of hyperoxia exposure, which can lead to misclassification of patients. Assuming the deleterious effect of exposition to supra-normal oxygen tension after cardiac arrest, precise time point when oxygen may have a detrimental effect remains unclear. Experimental evidence supports the assumption that early hyperoxia should be more pernicious than late hyperoxia [36] and that oxidant injury occurs rapidly after cardiac arrest [37]. However, results are different in humans. Indeed, one study evaluated the impact of PaO_2_ levels during cardiopulmonary resuscitation on cerebral performance status after hospital admission. In this study, 28 % of patients survived with cerebral performance category CPC 1 or 2 in the hyperoxia group (PaO_2_ during CPR >300 mmHg) whereas 23 and 14 % of patients survived with a CPC 1 or 2 in the normoxia and hypoxia group, respectively [32]. Nevertheless, these interesting results should be taken cautiously given the higher rate of hospital admission in the hyperoxia group (83.3 vs. 50.6 % in the normoxia group and 18.8 % in the hypoxia group) that could indicate a better initial prognosis of hyperoxic patients.

Recently, three meta-analyses pooled observational studies studying the relationship between hyperoxia and outcomes in post-cardiac arrest [38–40]. Hyperoxia appears to be correlated with increased intrahospital mortality. However, these results must be interpreted cautiously given the heterogeneity and the limited sample size in analyzed studies. Moreover, as highlighted by the authors, they reconstructed OR when not provided and some patients are overlapped within the population studied by Bellomo et al. [24] and Ihle et al. [30] using the ANZICS database.

## How to think out of the box?

Given their short life, ROS cellular damages require the postulate of an increased oxygen tension within the damaged tissue. However, arterial PaO_2_ is possibly not an accurate estimate of tissular oxygen delivery, particularly when cerebral blood flow is decreased. Using a swine model of cardiac arrest, Rossi et al. found a negligible improvement of cerebral oxygen consumption under hyperoxia condition during cerebral blood flow reduction mimicking CPR [41]. Therefore, a regional cerebral tissue oxygenation monitoring before and after ROSC could improve the comprehension of hyperoxia mechanisms.

Most of the clinical studies focus on intrahospital mortality or on cerebral performance status as the main outcome measure for hyperoxia exposure after cardiac arrest and hypothesize an increased ROS production-mediated process. However, outcome may be related to other mechanisms. For instance, although a causal role has never been highlighted, hyperoxia is recognized to induce pulmonary dysfunction and may affect patient outcome after cardiac arrest. Only one recent study investigated whether high levels of oxygen exposure after cardiac arrest contribute to pulmonary dysfunction. They found no relation between pulmonary compliance and higher exposure to oxygen in the first hours [42]. Nevertheless, whether hyperoxia may participate to ventilator-acquired pneumonia occurrence or could increase cardiac dysfunction remains unknown and should be investigated in further studies.

Even if oxidative stress is a well experimentally documented mechanism of toxicity in ischemia–reperfusion, evidence in the cardiac arrest context is scarce. Only one study reports decreased amounts of derivatives of reactive oxygen metabolites but also a decreased global antioxidant capacity of blood plasma after cardiac arrest under therapeutic hypothermia [43]. Unfortunately, baseline oxidative status is not compared to control population and therefore cannot be evaluated. Cardiac arrest is strongly supposed to induce ROS overproduction, but effects on antioxidant defenses are poorly documented, so as the levels of ROS production when compared to other populations of patients such as cerebral trauma or cerebral ischemia. Considerations on these aspects should be part of the analysis on hyperoxia toxicity after cardiac arrest and could therefore lead to reconsider and revisit the current concept of oxygen toxicity after cardiac arrest.

Although PaO_2_ provided by arterial blood gas is the only measurement of oxygen concentration in blood, dissolved oxygen is only responsible for ROS production. Dissolved oxygen quantification should therefore be the main parameter assessed in studies. Moreover, estimation of dissolved oxygen in blood should take into account temperature-related variation. For instance, decreasing central temperature from 37 to 33 °C is associated with a 2.7-fold increase in dissolved oxygen in the blood, which might increase oxidative damages.

## Conclusion

Cardiac arrest is a cataclysmic form of global ischemia–reperfusion affecting all organs, leading to reconsider the direct toxicity of hyperoxia on neurological outcome and mortality, considering ROS-mediated injuries observed in animal models. Many questions are still unsolved: Assuming an imbalance in oxidative stress equilibrium, is there a decrease in antioxidative defenses, an increase in ROS amounts or both? What is the role of nitrogen species? Does dissolved oxygen play a significant role?

Variability in primary outcome in human studies highlights the difficulties encountered by authors in examining outcome (Table 3). It is important to note that none of the animal studies establish any repercussion of hyperoxia on survival. Given the suspected pathophysiology of hyperoxia toxicity and the evidence from animal experimental studies, this problem emphasizes the importance of focusing also on neurological damages induced by high oxygen tension. We suggest that a well-designed prospective study should assess whether receiving increasing values of arterial oxygen partial pressure (using temperature-corrected oxygen dissolved in blood as a continuous variable), as soon as possible after ROSC, is or not associated with a difference in oxidative stress imbalance and outcome.

**Table 3:** Ongoing studies related to hyperoxia in the cardiac arrest context

Study	Title	Identifier	Location	Characteristics	Objective
EXACT study	Reduction of oxygen after cardiac arrest	NCT02499042	Australia	Prospective randomised controlled trial	Evaluate the feasibility of paramedic titration of oxygen delivery in adults resuscitated from cardiac arrest
REOX study	Reoxygenation after cardiac arrest	NCT01881243	USA	Prospective observational study	Testing the association between hyperoxia exposure after resuscitation from cardiac arrest and outcome and oxidative tress status
